# Functional Amyloids in Adhesion of Non-*albicans Candida* Species

**DOI:** 10.3390/pathogens14080723

**Published:** 2025-07-22

**Authors:** Melissa C. Garcia-Sherman, Safraz A. Hamid, Desmond N. Jackson, James Thomas, Peter N. Lipke

**Affiliations:** Department of Biology, Brooklyn College of the City University of New York, Brooklyn, NY 11215, USAsafraz.hamis@yale.edu (S.A.H.); djackson@brooklyn.cuny.edu (D.N.J.); jamesthomas824@gmail.com (J.T.)

**Keywords:** non-*albicans Candida*, *C. tropicalis*, *C. parapsilosis*, *C. krusei*, adhesins, Als family, fungal–host adhesion, functional amyloids

## Abstract

*Candida* fungal species are the most common fungal opportunistic pathogens. Their ability to form antifungal resistant biofilms contributes to their increasing clinical frequency. These fungi express surface-anchored adhesins including members of the Als family. These adhesins mediate epithelial adhesion, aggregation, and biofilm formation. Many of the adhesins contain cross-β core sequences that form amyloid-like protein aggregates on the fungal surface. The aggregates mediate high-avidity bonding that contributes to biofilm establishment and persistence. Accordingly, autopsy sections from individuals with candidiasis and other mycoses have amyloids within abscesses. An amyloid-forming peptide containing a sequence from *Candida albicans* Als5 bound to *C. albicans*, *C. tropicalis*, and *C. parapsilosis*. *C. albicans* and *C. tropicalis* aggregated with beads coated with serum albumin, and the aggregates stained with the amyloid-binding dye thioflavin T. Additionally, an Als5-derived amyloid-inhibiting peptide blocked cell aggregation. The amyloid-inhibiting peptide also blocked *C. albicans*, *C. tropicalis*, and *C. parapsilosis* adhesion to monolayers of FaDu epithelial cells. These results show the involvement of amyloid-like interactions in pathogenesis in several *Candida *species.

## 1. Introduction

*Candida *species are the most common cause of fungal infections [1,2,3]. These fungi are commensal, but they can also cause systemic, oral, and genital infections in immunocompromised patients. Although the majority of fungal infections are caused by *Candida albicans*, other *Candida *species are becoming more prevalent [1,4], including *C. tropicalis* and the *C. parapsilosis *complex. Another emergent pathogen is *Pichia kudriavzevii*, commonly called *Candida krusei*, the name we will use here. These fungi are often resistant to common antifungal drugs. For example, *C. parapsilosis* is now commonly associated with healthcare worker-transmitted nosocomial infections [3].

**Figure 1:**
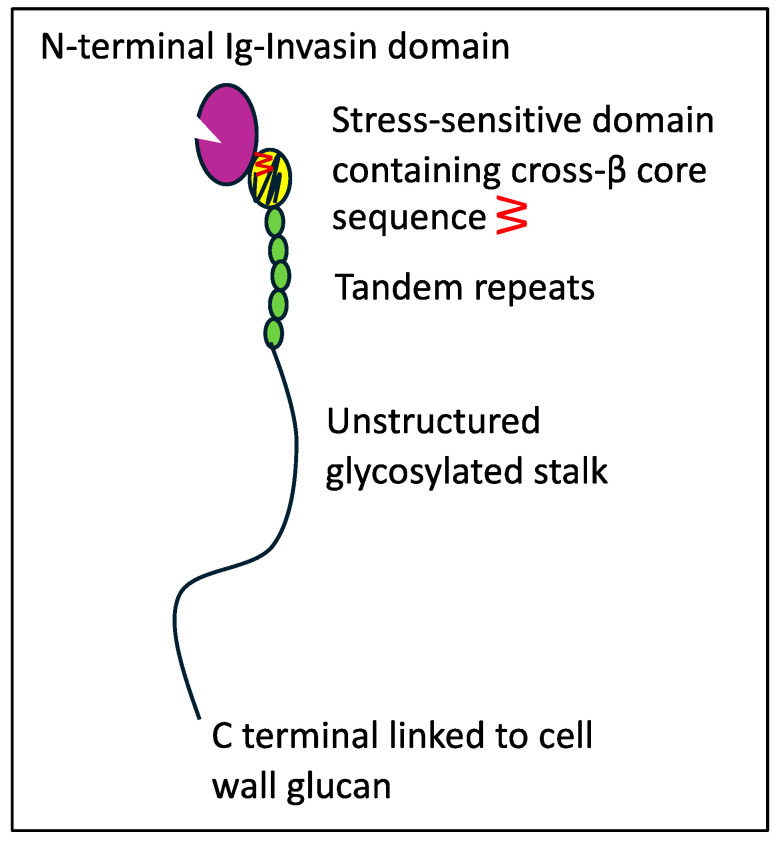
Graphic representation of an Als adhesin. The number of tandem repeats and the stalk length vary widely [22]. Reprinted with permission [19].

Adhesion and persistence are initial steps for infection [1,5,6]. *C. albicans *is the best studied exemplar. This opportunistic pathogen expresses dozens of adhesin genes from several gene families. The expression of each gene varies with the growth phase, fungal morphology, nutrition, and environmental signals [7,8,9,10]. Most of the adhesins are glycoproteins with N-terminal adhesion domains, short tandem repeats, long Ser/Thr-rich hyperglycosylated regions, and modified glycosylphosphatidylinositol (GPI) anchors covalently linked to wall glucan [5,10,11,12,13]. Adhesins in the Als family are encoded at eight loci in *C. albicans*. Each Als adhesin has two Ig-like invasin domains at the N-terminal (Figure 1, colored purple) [14,15,16]. This region binds C-terminal peptides, a common internal three-amino acid sequence motif, and L-fucose. The Thr-rich region (TR region, yellow) is the C-terminal to the second Ig-invasin domain. Following the T region is a variable number of 36-amino acid tandem repeats (TR region, green) [17], which participate in hydrophobic-effect adhesion and aggregation [18]. The C-terminal to the TR region, the Ser/Thr-rich hyperglycosylated stalk, consists of 700–1000 amino acids. The stalk allows motion around the cell wall attachment site and is thus critical for adhesin clustering on the cell surface [19,20,21].

The Als T region is highly conserved. It contains a sequence with >90% potential in the amyloid/cross-β predictor TANGO [19,20,21]. The cross-β core sequences are identical in Als1, Als3, and Als5, and highly similar in Als2, Als4, Als6, and Als9 [17,23]. Cross-β bonding has been demonstrated in Als5 and Als1, as well as in several non-homologous adhesins [19,20,21,24,25,26,27,28,29]. In Als1, Als3, and Als5, the sequence is ^322^SNGIVIVATT^331^. The sequence contains a three-residue β-turn motif followed by five consecutive hydrophobic aliphatic residues. It mediates functional amyloid formation resulting in adhesin clustering, high-avidity binding, and cell–cell adhesion. When Als5 is expressed in *S. cerevisiae*, the cross-β core sequence is necessary for yeast aggregation, cell–cell adhesion, and catch bonding [19]. Catch bonding is the ability to bind surfaces and to aggregate more tightly under shear stress. These activities mediate biofilm formation and persistence under flow.

The T-region cross-β core peptide SNGIVIVATTRTV, is designated as “Als-amyloid-binding peptide.” It forms fibers with an X-ray diffraction pattern characteristic of cross-β geometry [30]. This peptide when fluorescently labelled specifically stains cells expressing Als adhesins. Fluorescence is greatly attenuated in the *C. albicans als1/als1 als3/als3 *deletion strain. The fluorescent peptide also stains *C. albicans* colonies in clinical specimens including in autopsy sections from candidiasis victims. It does not stain human tissues or untransformed *S. cerevisiae*. A fluorescent peptide of the same composition but scrambled sequence does not stain Als-expressing cells (Supplemental Figure S1) [24].

A single amino acid substitution (V326N) within the T domain of the cross-β core sequence greatly reduces protein aggregation potential without affecting affinity to ligands that bind to the Ig-invasin region [21,24]. The peptide SNGINIVATTRTV (“Als-anti-amyloid peptide”) shows very low cross-β core potential. It fails to form amyloid fibers, and it inhibits adhesin clustering, cell–cell binding, and biofilm formation. The defect in cells expressing Als5^V326N^ adhesin can be corrected by addition of excess Als-amyloid-binding peptide [21,24]. The Als-anti-amyloid peptide acts as a sequence-specific anti-adhesin for Als family adhesins [20,21,26].

Cross-β core sequences are extremely common in fungal adhesins. Their functionality has been demonstrated in several adhesin families in *C. albicans* and *Saccharomyces cerevisiae* [19]. Fungal abscesses in autopsy sections from patients with mycoses, including candidiasis, bind amyloid dyes [19,24,31,32,33]. Histochemistry shows that the fungal cells in the abscesses bind Serum Amyloid P Component (SAP), a marker of amyloids [31,34,35,36,37,38]. These data support the existence of amyloid-like interactions within a broad range of fungal abscesses during infection.

Als family adhesins within the CUG clade of *Candida* show high sequence conservation of the cross-β core sequence [10,22,39,40]. The *C. tropicalis* genome contains 13 Als homologous loci, and *C. parapsilosis *has 5. In each case, the T region is highly conserved. In all but a few cases, the cross-β core sequence is conserved. There are functional data supporting the expression, activity and role in pathogenesis of Als adhesins in both *C. tropicalis *and *C. parapsilosis* [41,42,43,44,45,46,47,48,49,50,51,52]. We assayed to determine whether the Als-amyloid-binding peptide can be used as a probe to identify amyloid-like interactions on the cell surface of three non-*albicans* species, *C. tropicalis*, *C. krusei*, and *C. parapsilosis*. We also tested the Als-anti-amyloid peptide in fungal cell aggregation and adhesion to oral epithelial cells. Collectively, the results indicate that amyloid-like interactions function in yeasts that express Als family adhesins.

## 2. Materials and Methods

### 2.1. Yeast Strains and Growth Conditions

Yeast strains included *Candida tropicalis* (ATCC 28707), *Candida krusei* (ATCC 62578), *Candida parapsilosis* (A-71), and *C. albicans *DAY286. Non-*albicans Candida* strains were streaked from frozen stocks onto YPD agar (2% glucose, 2% peptone 1% yeast extract 2% agar). *C. albicans* DAY286 was grown weekly on YPD agar supplemented with 80 mg/L uridine. Streaked plates were stored at 18 °C and used for no longer than two weeks. Unless otherwise indicated, overnight cultures were grown at 30 °C in YPD liquid medium with shaking at 170 rpm overnight (18 h). Cell numbers were determined by spectrophotometry (ThermoSpetronic 20 DX, Thermo Fisher Scientific, Waltham, MA, USA) at 600 nm.

### 2.2. Cell Lines and Growth Conditions

The FaDu pharyngeal carcinoma cell line was obtained from ATCC (Manassas, VA, USA). Cells were cultured at 37 °C with 5% CO_2_ in Minimal Essential Medium (MEM) (Invitrogen, Grand Island, NY, USA) supplemented with 1 mM sodium pyruvate, 1× non-essential medium), 100 U penicillin and streptomycin (all from Invitrogen), and 10% FBS (Atlanta Biologicals, Flowery Branch, GA, USA) (CMEM). Cells were grown to sub-confluency and passaged. Passages 3–12 were used for these experiments. All cell counts were performed using a hemacytometer.

### 2.3. Amyloid Peptide Detection

Als-amyloid-binding peptide SNGIVIVATTRTV and Als-anti-amyloid peptide SNGINIVATTRTV were synthesized (Lifetein, Somerset, NJ, USA).The Als-amyloid-binding peptide was labeled with fluorescein as previously described [24]. Briefly, the peptide was incubated and mixed overnight at pH9 with fluorescein isothiocyanate (FITC) adsorbed onto celite (Sigma Chemical Co., Saint Louis, MO, USA). The celite and unreacted FITC were removed by centrifugation. The purity and composition of the labelled peptide were confirmed by HPLC-MS analysis. Liquid cultures of *Candida *species were prepared using 20 mL of YPD media and inoculating overnight at 30 °C. A total of 1 × 10^7^ cells were washed three times in 3 mL of phosphate-buffered saline (PBS, pH 7.4: 10 mM sodium phosphate, 137 mM NaCl, 2.7 mM KCl). Fluorescein-labeled Als-amyloid-binding peptide (20 μg/mL) or PBS only was added to the cells and incubated at room temperature on a horizontal shaker for 30 min. The cells were then washed two times in 1 mL of PBS buffer solution and viewed using an Olympus BX51 fluorescence microscope (Olympus Corp. of America, Center Valley, PA, USA). The cells were examined under both bright-field and fluorescein filter sets (494 nm excitation and 518 nm emission). The exposure under the fluorescent filter was maintained at 1/12 s.

### 2.4. In Silico Analysis

The Als5 amino acid sequence (GenBank AF025429.1) was entered into the *Candida* genome database (www.candidagenome.org), accessed on 31 March, 2015, and a BLASTP 2.9.0 was performed against the entire *C. tropicalis *and *C. parapsilosis* genome. A BLAST search (www.ncbi.nlm.nih.gov) of the *Pichia kudriavzevii* (*C. krusei*) database (TaxID: 4909) was also carried out, using amino acid sequences from *C. albicans* Als5, *C. glabrata* Epa1 (GenBank ALG76051.1), and *S. cerevisiae *Flo11 (GenBank P08640.2) as queries. The BLAST alignment of residues 322–331 of hits that had an *e* value smaller than 1.0 were examined for homology with the Als-amyloid-binding peptide SNGIVIVATT. The sequences were examined using TANGO (tango.crg.es, accessed on 15 April, 2015) analysis for potential to form beta-sheet aggregation. Beta-sheet aggregation percentages above 5% were considered positive for beta aggregation [53].

### 2.5. Aggregation Assays

Aggregation assays were previously described [23]. Briefly, *Candida *cells were grown in YPD overnight at 30 °C. The cells were washed twice with 1× TE buffer (10 mM Tris-HCL, 1 mM EDTA) with centrifugation at 3000 rpm for three minutes between each wash. Then, 1 × 10^6^ heat denatured BSA-coated magnetic beads were added to 1 × 10^8^ cells. In Als-anti-amyloid peptide inhibition experiments, 200 µg of Als-anti-amyloid peptide was added to the assay. In experiments where thioflavin T staining was performed, 100 nM of the dye was added [24]. Water was used as a vehicle for both peptide and thioflavin T. Vehicle control samples are presented for comparison. The aggregations were incubated at 24 °C for 45 min with shaking at 100 rpm. Aggregates were separated out using a magnet and washed on the magnet with 500 μL 1× TE buffer. An extra wash was performed when staining with thioflavin T to remove excess dye. Aggregates were resuspended in 100 μL of 1× TE buffer. The sample (25 μL) was wet-mounted on a slide and observed by microscopy. Thioflavin T fluorescence was observed using the Olympus BX51 425 nm excitation and 510 nm emission filter set.

### 2.6. FaDu Monolayer Adhesion Assays

Adhesion assays were performed according to a previously described protocol with the following modifications [54]. To generate monolayers 1 × 10^5^ FaDu cells in CMEM were plated on Corning^®^ BioCoat fibronectin-coated 22 mm round coverslips (Thermofisher, Waltham, MA, USA), housed in a 6-welled polystyrene tissue culture plate, and grown to confluency. Overnight cultures of each yeast strain were washed 3 times in ddH_2_O, and 3 × 10^6^ cells in 3 mL of CMEM were incubated for 1 h at 37 °C with shaking at 100–170 rpm. After incubations, the yeast cultures were added to the FaDu monolayers and incubated for 3 h at 37 °C in 5% CO_2_ (with or without Als-*anti*-amyloid peptide, 20 µg/mL). Monolayers were washed 3 times with calcium and magnesium-free Hank’s balanced salt solution (Hyclone, Logan, UT, USA) and fixed in 4% paraformaldehyde for 1 h at room temperature. Fixed monolayers were washed 4 times with 1× Dulbecco’s phosphate-buffered saline (DPBS) (Corning^®^-Thermofisher, Waltham, MA, USA). Monolayers were stained with 25 µg/mL Texas Red-Con A (Molecular Probes-Thermofisher, Waltham, MA, USA ) for 1 h at room temperature and washed 2 times with DPBS. The FaDu cells within the monolayers were permeabilized for 30 min with 0.5% Triton X-100 in DPBS. The monolayers were washed once with DPBS; then, 0.01 mg/mL of calcofluor white in PBS was added for 10 min and washed two more times with PBS. Stained monolayers were observed using a Nikon Eclipse 90i confocal microscope (Nikon Corp., Belmont, CA, USA) using settings for Texas red (561 nm excitation and 570 nm emission) and calcofluor white (408 nm excitation and 450 nm emission).

## 3. Results

### 3.1. Als-Amyloid-Binding Peptide Binds to Non-albicans Candida Species

**Figure 2:**
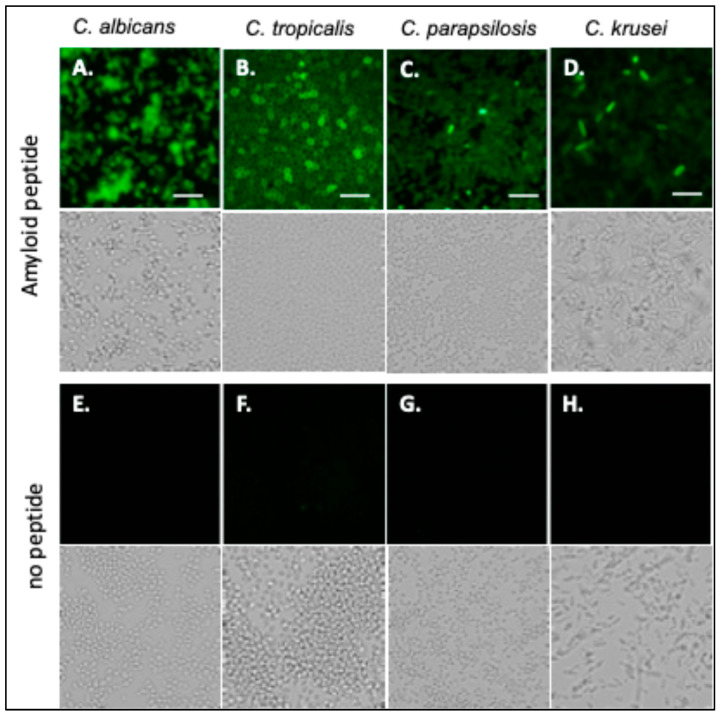
*Candida *species probed with Als-amyloid-binding peptide. (**A**) *C. albicans*, (**B**) *C. tropicalis* (**C**) *C. parapsilosis*, and (**D**) *C. krusei* probed with 20 µg/mL of fluorescein-labeled Als-amyloid-binding peptide. (**E**–**H**) Auto-fluorescence controls. Bright field images are positioned below fluorescent images. The scale bar represents 40 µm. The data shown are representative of a minimum of 3 experiments.

Fluorescein-labeled Als-amyloid-binding peptide specifically labels Als-expressing cells, including *C. albicans* [24]. *C. tropicalis*, *C. parapsilosis,* and *C. krusei* were also probed with fluorescein-labeled Als-amyloid-binding peptide, 20 µg/mL. *C. albicans*, *C. tropicalis*, and *C. parapsilosis* bound the peptide well (Figure 2A–C). In the *C. krusei* culture, about 5% of the cells were brightly stained, but most cells stained very poorly (Figure 2D). Autofluorescence was not significant (Figure 2E–H).

### 3.2. C. tropicalis Aggregates Are Thioflavin T Positive and Inhibited by the Als-Anti-Amyloid Peptide

**Figure 3:**
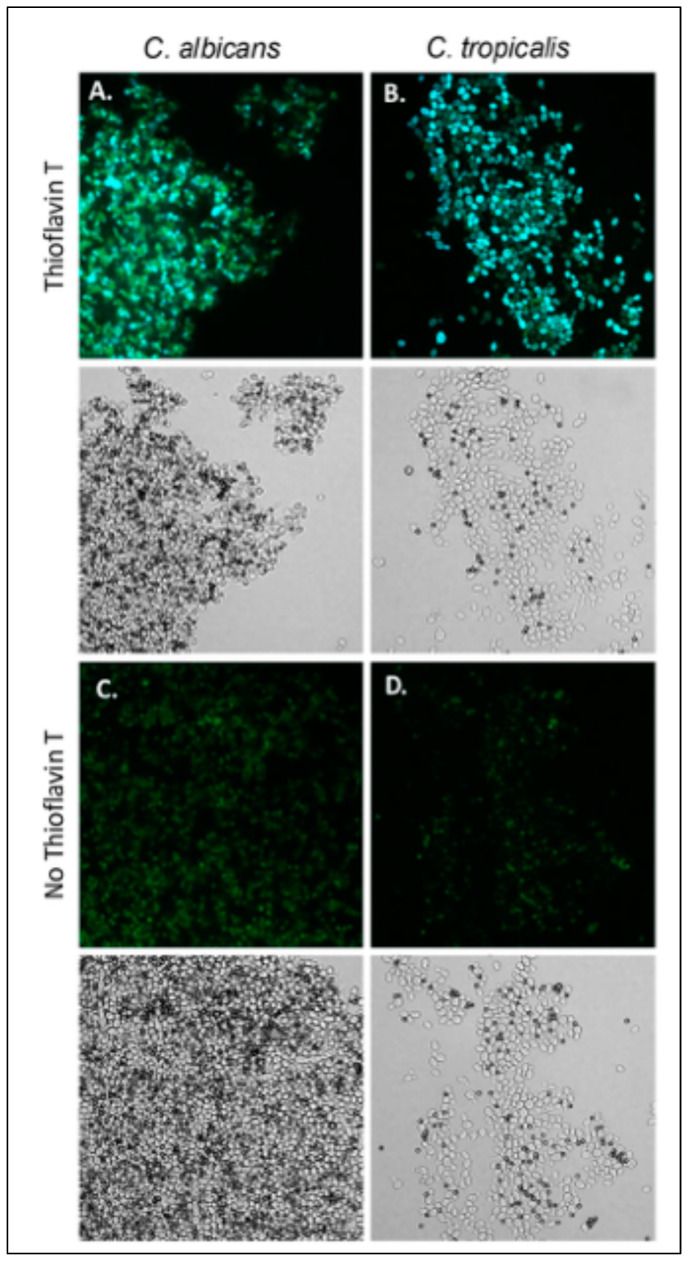
*C. tropicalis* aggregates bind thioflavin T. (**A**) *C. albicans* and (**B**) *C. tropicalis* aggregates stained with 100 nM thioflavin T. (**C**,**D**) Aggregates without addition of thioflavin T. Bright field images are below the fluorescence. The diameter of the beads (dark spheres) is 1 μm. The data shown are representative of four experiments.

*C. albicans* aggregates well with ligand-coated magnetic beads, and *S. cerevisiae* expressing Als5 and Als1 also does [20,21,23,24,55]. The aggregation is highly enhanced by amyloid interactions [20,21,25,56]. We assayed *C. tropicalis*, *C. parapsilosis,* and *C. krusei* for the ability to aggregate (Figure 3)). Of these strains, *C. tropicalis* aggregated in the presence of beads coated with fibronectin or heat-denatured BSA. *C. tropicalis* aggregates were less compact than *C. albicans* aggregates (Figure 3A,B). The aggregates fluoresced brightly when stained with the amyloid dye thioflavin T dye, 100 nM. This result demonstrated the presence of amyloid-like interactions on the surface of aggregated *C. tropicalis*.

**Figure 4:**
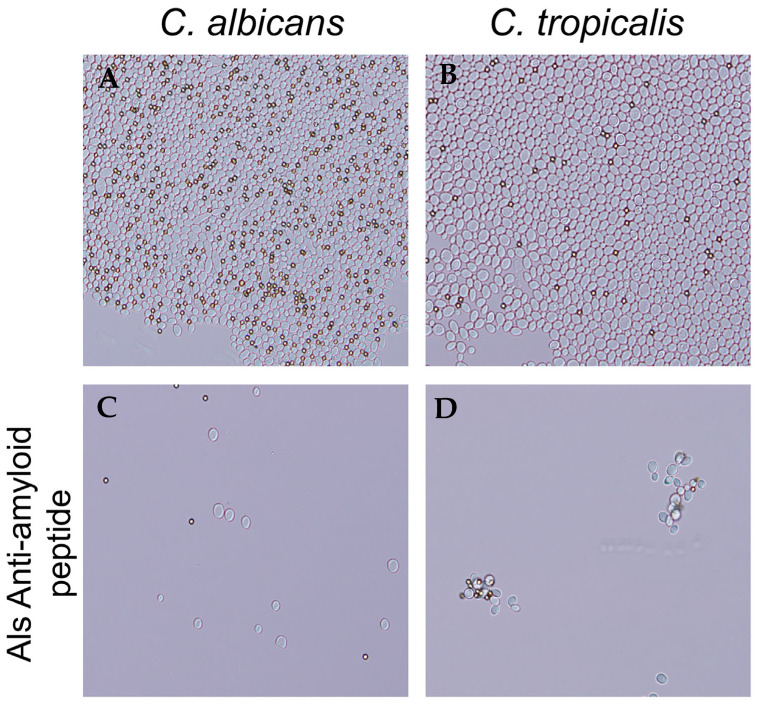
Als-anti-amyloid peptide blocks *C. albicans* and *C. tropicalis* cell aggregation. (**A**) *C. albicans* and (**B**) *C. tropicalis* aggregates without addition of peptide. (**C**) *C. albicans* and (**D**) *C. tropicalis* aggregates formed in the presence of 200 µg/mL of Als-anti-amyloid peptide. The diameter of the beads (dark spheres) is 1 μm. Data shown are representative of three experiments.

In *C. albicans* adhesins Als5 and Als1, a V326N substitution in the amyloid-forming cross-β core sequence significantly decreases aggregation [21,25]. Additionally, the Als-anti-amyloid peptide blocks aggregation. We therefore hypothesized that the anti-amyloid peptide would block *C. tropicalis* cell aggregation. We aggregated cells in the presence and absence of 200 μg/mL of anti-amyloid peptide. The peptide prevented the formation of aggregates in *C. tropicalis*, as it does for *C. albicans* (Figure 4).

### 3.3. The Als-Anti-Amyloid Peptide Inhibits Adhesion to Oral Epithelial Cells

**Figure 5:**
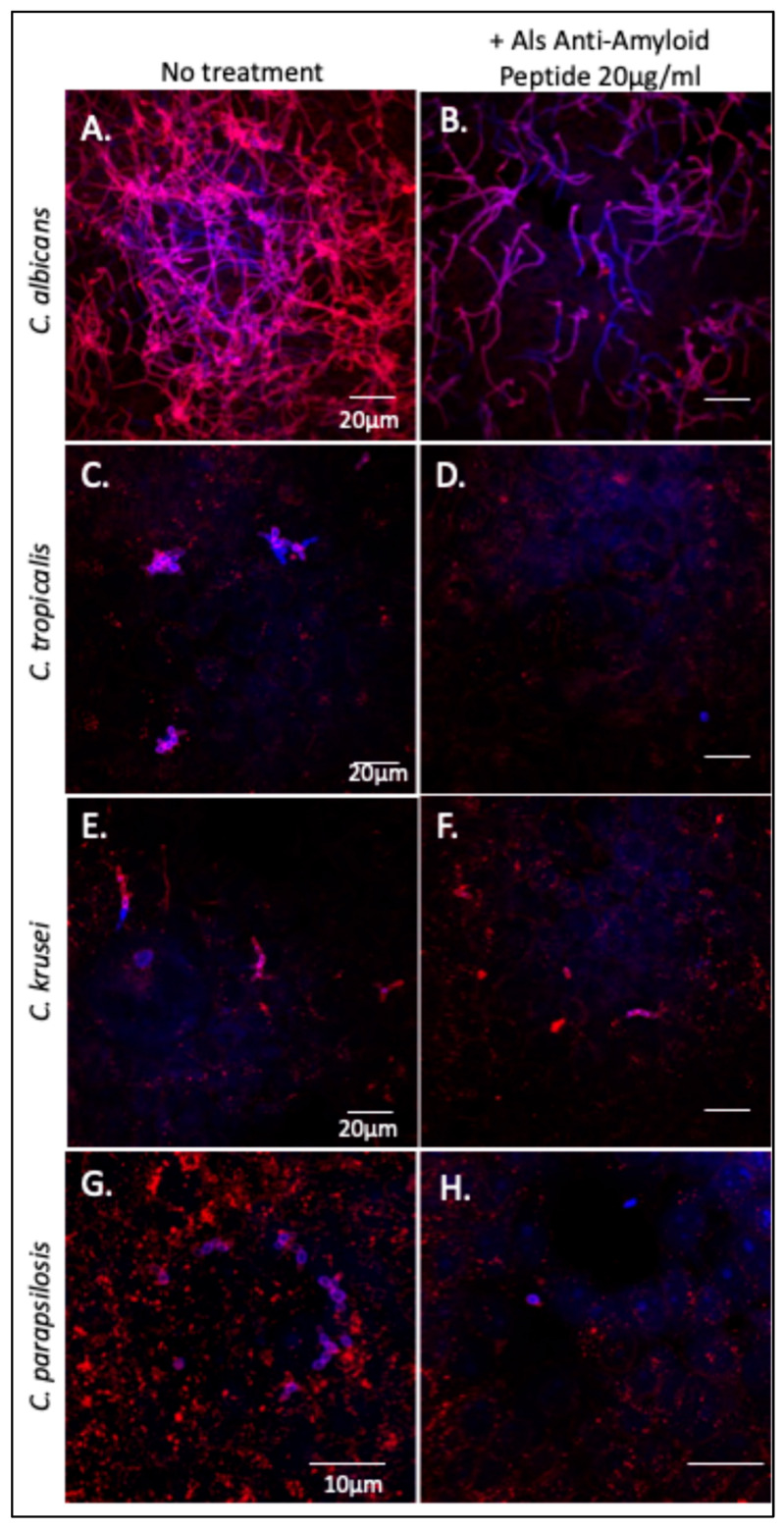
Effect of Als-anti-amyloid peptide on binding of *Candida *spp. to FaDu monolayers. Confocal micrographs of (**A**) *C. albicans*, (**C**) *C. tropicalis* (**E**) *C. krusei*, and (**G**) *C. parapsilosis* adhered to FaDu epithelial cell monolayers. Panels (**B**,**D**,**F**,**H**): *Candida* strains bound to monolayer in the presence of 20 µg/mL of Als-anti-amyloid peptide. Superficial fungi were stained with Texas red-labeled Concanavalin A (red). FaDu cells were permeabilized and then counterstained with the fungal stain calcofluor white (blue). Fungi within the monolayer cells are violet-blue, and those that are superficial are pink. The data shown are representative of two experiments.

The Als-anti-amyloid peptide inhibits cellular aggregation of *C. albicans* and *C. tropicalis* as well as substrate attachment and biofilm formation in *C. albicans* [21,24,26]. We tested whether this peptide would affect *Candida* spp. binding to and invasion of oral epithelial cells. *C. albicans*, *C. tropicalis*, *C. parapsilosis*, and *C. krusei* were each incubated with oral epithelial cell monolayers in the presence or absence of the Als-*anti*-*amyloid *peptide, 20 µg/mL. After incubation, the monolayers were extensively washed then stained with Texas-red-labeled Concanavalin A, which binds to mannoproteins on the fungal cell surface. The monolayers were washed again, permeabilized, and then co-stained with calcofluor white to visualize intracellular fungi. As expected, *C. albicans* formed a network of hyphae that were both superficially located (pink) and intracellular (violet-blue) (Figure 5A). Cultures treated with Als-anti-amyloid peptide showed >80% reduction of *C. albicans* binding to the FaDu cells (Figure 5B). There were also fewer intracellular fungi. However, the fraction of invading cells was similar in the presence and absence of peptide. Therefore, Als-anti-amyloid peptide blocked adhesion to the monolayer, but it did not alter the frequency of invasion.

*C. tropicalis* formed pseudohyphae that bound and invaded the monolayers (Figure 5C,E). The Als-anti-amyloid peptide (20 µg/mL) blocked binding of *C. tropicalis* by ~90% (Figure 5F,G). As for *C. albicans*, there were fewer intracellular fungi, but the peptide did not block monolayer invasion.

*C. parapsilosis* did not form hyphae or pseudohyphae. The yeast form bound to FaDu monolayers (Figure 5G), and the Als-anti-amyloid peptide inhibited binding to the monolayers (Figure 5H). As with *C. albicans*, the bound fungi invaded the FaDu cells with similar frequency in the presence and absence of Als-anti-amyloid peptide. The peptide did not noticeably affect *C. krusei* binding to the monolayers. Together, these data indicate that the Als-anti-amyloid peptide can interfere with *C. albicans*, *C. tropicalis* and *C. parapsilosis* binding to FaDu monolayers.

## 4. Discussion

The Als adhesins of *C. albicans *have multiple functions, including yeast aggregation, biofilm formation, and immune modulation [5]. Functional amyloid-forming cross-β core sequences cluster these adhesins on the cell surface. The result is catch-bonding, high-avidity binding to substrates, and strong cellular aggregation. These activities lead to biofilm establishment, persistence, and immune modulation [19]. Similar functions for cross-β core sequences are known for several other fungal adhesins, including *C. albicans* Pga59 and *S. cerevisiae* Flo1 and Flo11 [20,26,27]. Our data extend these observations to functional amyloids in non-*albicans Candida* species. In both *C. tropicalis *and *C. parapsilosis *Als adhesins are expressed on the cell surface [39]. In both species, adhesins are important for adhesion, biofilm formation, and pathogenesis [44,45,48,49,50].

### 4.1. The Als5-Amyloid-Binding Peptide as a Probe

The Als5-amyloid-binding peptide specifically binds to *C. albicans* yeast and hyphae. The peptide also binds to *S. cerevisiae* that express Als5 or Als1 [24,31]. Our results show similar staining of *C. tropicalis *and *C. parapsilosis*. All the cells of each species bound the peptide, and in each case, a subpopulation stained brightly. The binding was consistent with the presence of Als family adhesins in each species (Supplementary Tables S1 and S2) [10,22,40].

In *C. krusei*, peptide staining was dim for most cells, with ~5% of cells staining as brightly as some *C. albicans *or *C. tropicalis *cells. Because no Als adhesins have been found in the genome of this fungus, this staining was not consistent with the previously demonstrated specificity of peptide binding. However, *C. krusei* expresses many adhesins from other families [57]. Several of these adhesins include non-homologous cross-β core sequences with sequential aliphatic hydrophobic residues, like the Als-amyloid-binding peptide itself (Supplementary Table S3). These regions might bind the probe peptide through hydrophobic effect, even in the absence of sequence specificity. Thus, the data show that although Als-amyloid-binding peptide has high affinity for Als family cross-β core sequences, binding was not completely specific.

### 4.2. Als T Region Cross-β Core Sequences in Non-Albicans Candida

There are 13 *ALS* loci in *C. tropcalis* (*CtAls* loci) and 5 in *C. parapsilosis *(*CpAls* loci) (Tables S2 and S3) [10,22,40]. These loci encode adhesins with conserved T domains. All but three of these contain the conserved cross-β core sequence in the same position as in the *C. albicans* adhesins. The cross-β core sequences are located approximately 24 residues C-terminal to the last Cys residue in Ig-invasin domain II.

**Figure 6:**
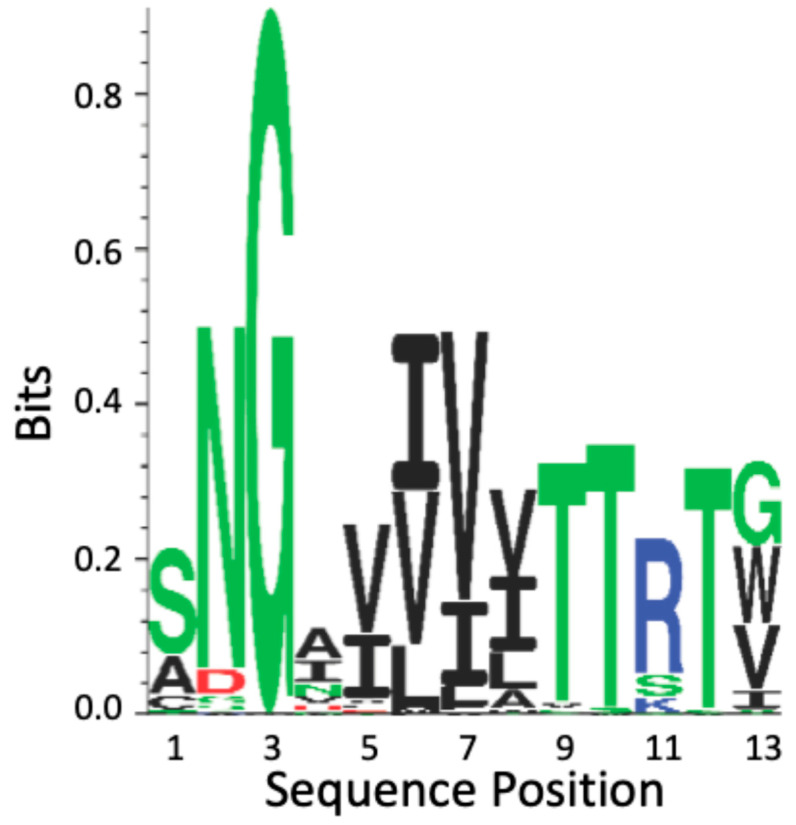
Sequence conservation of the cross-β core sequence in the T domain of *Candida* Als adhesins. The profile is based on amino acid occurrence in the 20 Als adhesin sequences from *C. albicans*, *C. tropicalis*, and *C. parapsilosis* (Figure S2), and all amino acids represented in the figure are listed there. The amino acids are color-coded by side chain properties: green represents polar residues; black, hydrophobic; red, acidic; and blue, basic. The height of each character is proportional to its selection coefficient at that position [58].

The CtAls T region cross-β core sequences show high conservation; they are 100% similar and 80% identical (Figure 6 and Figure S2). The regions flanking the CtAls cross-β core sequences are 84% identical to the Als-amyloid-binding peptide. This degree of conservation is significantly greater than the conservation levels in the N-terminal Ig-invasin domains of the proteins [40,58].

All the CpAls proteins have positionally conserved homologous sequences in the T domain. However, these sequences differ from those in *C. albicans* and *C. tropicalis *in that they substitute Ala for Ser in the first position and Trp or Ile instead of Val or Gly in the last position. The β-aggregation potential for CpAls adhesins is lower than in the other two species. In fact, one of these sequences (CpAls4800) has a TANGO β-aggregation value of only 6–9%. In conclusion, the CtAls and CpAls adhesins show conservation of the cross-β core, both in sequence and in position in the protein.

### 4.3. Activity of the Als-Anti-Amyloid Peptide

In contrast to the non-specificity of the Als-amyloid-binding peptide, the Als-anti-amyloid peptide only affected cells that have Als family adhesins. In *C. albicans *the Als-anti-amyloid peptide inhibits formation of cross-β bonds that cluster Als adhesins on the cell surface. Consequently, the Als-anti-amyloid peptide prevents avid binding to ligands, and it also prevents cell–cell binding through cross-β bonds. The activity of this peptide against *C. tropicalis* was fully consistent with that model. Both *C. albicans *and *C. tropicalis *aggregated with beads coated with heat-denatured BSA (Figure 2). The aggregates stained brightly with the amyloid dye thioflavin T. As with *C. albicans*, the Als-anti-amyloid peptide (200 µg/mL) was a potent inhibitor of aggregation. Furthermore, at a concentration 10-fold lower, the Als-anti-amyloid peptide inhibited binding of both species to FaDu oral epithelial cells by >80% (Figure 4). This concentration of the Als-anti-amyloid peptide also greatly inhibited *C. parapsilosis* binding to the epithelia. In contrast, this peptide did not inhibit binding of *C. krusei *(*Pichia kudriavzevii*), which does not express Als-family adhesins. The lack of effect of the Als-anti-amyloid peptide against *C. krusei *is consistent with its specificity for Als family adhesins.

### 4.4. Conclusions

There is extensive data showing that *C. albicans *Als adhesins form amyloid-like cross-β bonds. These bonds cluster the adhesins on the cell surface to mediate fungal cell aggregation, as well as biofilm formation and biofilm persistence. The data reported above confirm the involvement of amyloid-forming Als adhesins in *C. albicans*, *C. tropicalis*, and *C. parapsilosis. *Each of these fungi expresses Als adhesins with conserved cross-β core sequences in the T region. These sequences had similar activities in the aggregation of *C. tropicalis* and *C. albicans* [20,21]. Furthermore, in all three organisms, the Als-anti-amyloid peptide inhibited cross-β interactions. The consequence of this was greatly attenuated binding to oral epithelial cells and subsequent cell invasion. In conclusion, Als adhesin cross-β aggregation promotes binding to the host, biofilm formation, and pathogenesis in each of these *Candida *species.

## Data Availability

Data is contained within the article and Supplementary Material. Further inquiries can be directed to the corresponding author.

## Supplementary Materials

The following supporting information can be downloaded at: https://www.mdpi.com/article/10.3390/pathogens14080723/s1, Figure S1: *S. cerevisiae cells* stained with fluorescent peptides. Figure S2: Sequence alignment used to generate Figure 6. Table S1: Analysis of *C. tropicalis* proteins for homology to Als5 and putative amyloid-forming sequences utilizing TANGO and BLASTP. Table S2: BLASTP and TANGO analysis of *C. parapsilosis* proteins for homology to Als5 and putative amyloid-forming sequences. Table S3: TANGO-positive sequences in some putative adhesins in *C. krusei*.
