# Supplementary material for: Functional Amyloids in Adhesion of Non-albicans Candida Species
Source: Pathogens. 2025 Jul 22;14(8):723. doi: 10.3390/pathogens14080723 (PMC12389486; doi:10.3390/pathogens14080723)
Supplement: Supplementary file 1 [file pathogens-14-00723-s001.zip › pathogens-3739992-supplementary.pdf]

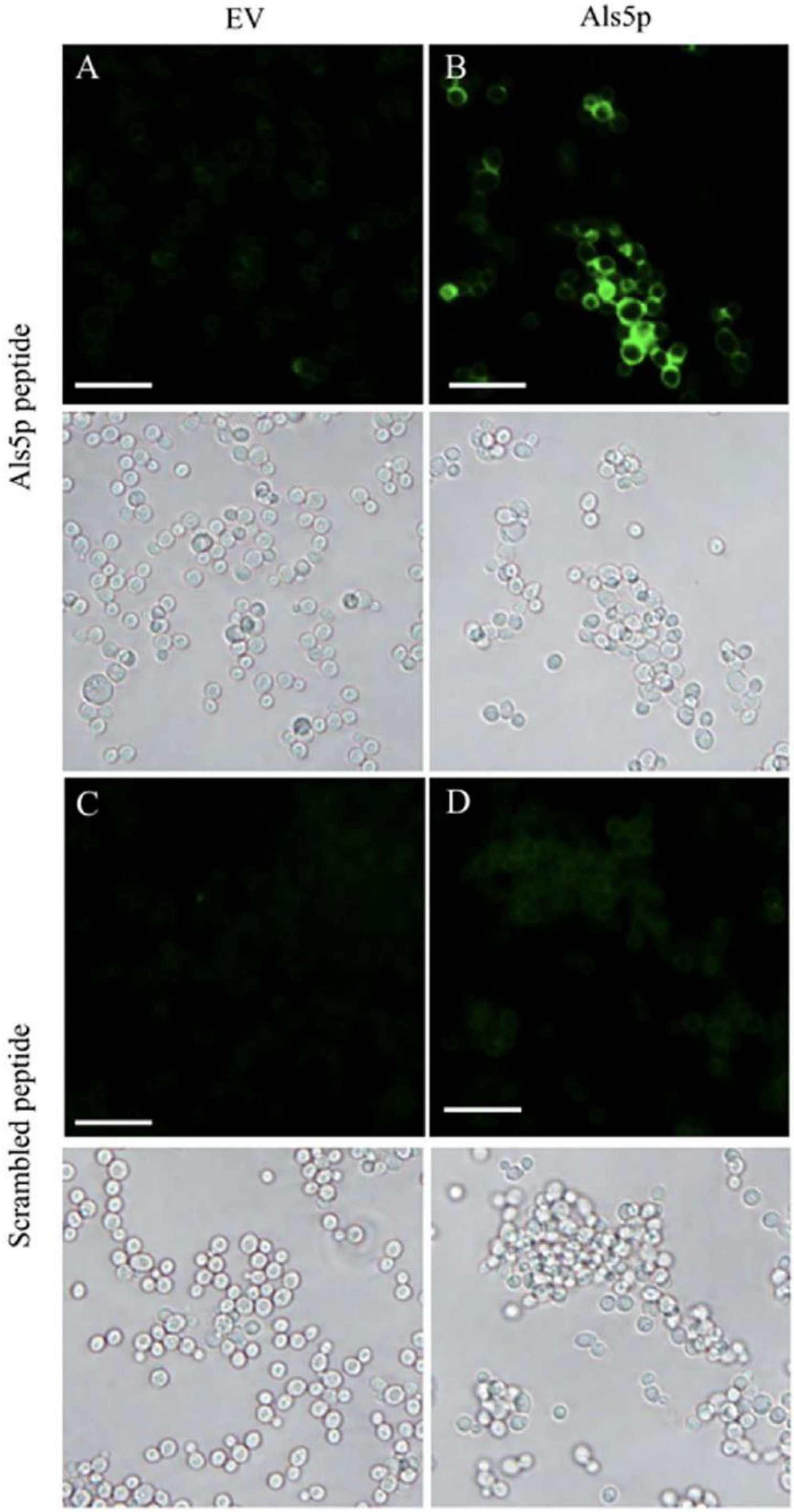

Figure S1. Als5p expressing *S. cerevisiae* cells stained with fluorescent peptides. *S. cerevisiae* harboring an empty vector (A,C) or expressing Als5 (B, D) were stained with amyloid peptide (200 mg/ml; A, B) or a scrambled-sequence peptide of identical composition (200 mg/ml; C, D). Lower micrographs are bright field images. All scale bars are 30 mm. Reprinted with permission [23].

Species	Name	1st res.	Sequence
C. parapsilosis	Als4770	331	ANGNEVVVTTSTW
	Als4780	331	ANGNEVVVTTSTW
	Als4790	331	ANGIIVVLTTSTI
	Als4800	331	ANGNEVILTTSTW
C. albicans	Als1	322	SNGIVIVATTRTV
	Als3	322	SNGIVIVATTRTV
	Als5	322	SNGIVIVATTRTV
	Als9	325	SNGDIIVVTTKTV
	Als6	325	SNGAAVVVTTRTV
	Als2	322	SNGITIVVTTRTV
	Als4	321	SNGFVIVATTRTV
C. tropicalis	Als1028	324	SDGAVVFVTTSTY
	Als1038	321	SNGAILVITTRTG
	Als1041	324	SNGAILVITTRTG
	Als2228	325	SNGAVVVYTTSTV
	Als2293	323	SNGAVVVLTTITG
	Als3791	323	SNGAVVVLTTITG
	Als3871	324	CSGVVLVITTRTG
	Als3882-1	321	SSGDVIVITTRTG
	Als1030	322	SGDGAIIIVTTRT

Figure S2. Sequence alignment used to generate Fig. 6.

Protein	Protein Description*	BLASTP E value	Sequence	Position	Beta Aggregation
CtrAls1028	Putative ALS adhesin	2.0e-162	SDGAVVFVTTST	324-337	82
CtrAls3786	Putative ALS2p ortholog	2.0e-155	SNGAVVVLTT	323-332	90
CtrAls2293	Putative ALS adhesin	1.0e-154	SNGAVVVLTT	323-332	90
CtrAls0941	Putative ALS adhesin	6.0e-153	SYGQPIEIIT	323-332	0
CtrAls3871	Putative ALS adhesin	5.0e-144	CSGVVLVITT	323-332	95
CtrAl3882	Putative ALS adhesin	2.0e-141	SSGDVIVITT	321-331	91
CtrAls2229	Putative ALS6p ortholog	2.0e-137	SGGIMIPVTRT	327-336	0
CtrAls3797	Putative ALS adhesin	1.0e-136	SEGMEIVVATT	321-331	11
CtrAls3791	Putative ALS adhesin	1.0e-126	SNGAVVVLTT	323-332	96
CtrAls1030	Putative ALS adhesin	5.0e-126	GDGAIIIVTT	323-332	88
CtrAls1041	Putative ALS adhesin	3.0e-124	SNGAILVITT	324-333	60
CtrAls1038	Putative ALS adhesin	3.0e-119	SNGAILVITT	321-330	80
CtrAls2228	Putative ALS7p ortholog	6.0e-95	SNGAVVVYTT	325-334	75

Table S1. Analysis of *C. tropicalis* proteins for Als5 homology and putative amyloid-forming sequences utilizing TANGO and BLASTP. *Determined by CGC and NCBI. Underlined amino acids are TANGO positive.

Protein	Protein Description	BLASTP E value	Sequence	Position	Beta Aggregation
CpAls4790	Putative ALS6p ortholog	9e-154	ANGIIVVLTT	331-340	98
CpAls4800	Putative ALS7p ortholog	4.0e-125	ANGNEVILTT	332-341	0
CpAls4770	Putative ALS3p ortholog	1.0e-96	ANGNEVVVTT	330-339	13
CpAls4780	Putative ALS1p ortholog	6.0e-89	ANGNEVVVTT	331-340	11

Table S2. BLASTP and TANGO analysis of *C. parapsilosis* proteins for Als5 homology and putative amyloid-forming sequences. Protein description determined by CGC. Underlined amino acid sequences are TANGO positive.

Table S3. TANGO-positive sequences in some putative adhesins in *C. krusei.* Sequences with β-aggregation potential > 5 are listed. Sequences with 4 or more contiguous hydrophobic aliphatic residues are highlighted in blue. Two paralogs, KGK36234.1 and KGK36237.1, are separately color coded in orange or purple where the sequences differ.

Protein	Protein Description	BLASTP E value	Sequence	Position	TANGO Beta Aggregation score
KGK39255.1 (1496 aa)	Epa1p homolog: Hypothetical protein with PA14 domain	6.00E-13	VWSIT	25-29	8.35
			MVNVLVELKGFYYA	110-123	42.1
			SYVFLE	164-169	14.42
			VVYVNV	178-183	86.1
			MSFSVSTSVVIGA	224-235	25.6
			TTYYVT	269-274	9.2
			YSAVVVVV	295-302	70.5
			AMVWF	422-426	31.4
			GALFFTT	447-453	24.5
			VVLVNMLLSSVL	473-484	92.9
			YVISI	502-506	7.6
			TIATTITW	564-571	13.9
			IVTVYV	596-601	39.3
			SLFVGLAALLLA	1484-1495	83.9
KGK36234.1 (1305 aa)<br>KGK36237.1 (1299 aa)	Epa1p homologs: Hypothetical proteins with PA14 domains	5.0 E-7<br>8.0 E-7	VLTSIV	27-32<br>21-26	14.2<br>12.2
			AMVFIG	134-139<br>128-135	17.9<br>30.6
			ISWVYL	174-180<br>169-174	25.5
			IKIVYVNVISAGGIELLV	188-205<br>189-199	55.5
			VFQLV	219-223	6.9
			MVFQLV	212-217	48.5
			ATTFIY	242-247<br>236-341	10.1
			YTTTVI	266-270<br>260-265	10.9
			TIVSYVFG	283-290	70.3
			YFISTI	321-326<br>315-320	28.6<br>28.8
			SIIIIE	352-357	84.2
			VLLVIP	394-399<br>388-393	11<br>11.2
			LVTYSSFI	401-408<br>395-402	10.2<br>10.4
			TTVFTV	413-418	40
			YYTSTYLTTV	448-457	15.5
			VSYTTIVGVY	474-483	70.2
			TATFIAVTVV	507-516<br>501-510	78.3
			SVYYVVV	538-544<br>532-538	86
			WNYIVYIP	576-583	46
			GWNYIVYIP	569-577	41.7
			VLTSV	617-621	6.6
			ITSTLYY	600-606	8.3
			AITSTLYY	593-600	7.9
			VTGFQVI	611-617<br>605-611	9.9
			VLVTSV	617-621	6.6
			SIVTTTIT	679-686<br>673-680	26.7<br>26.4
			IVYVP	693-697	6.2
			STMLIFTTTT	755-764<br>749-758	42.5<br>42.8
			INIVV	790-794	6.1
			VVTKSIDIVVKSI	791-803	43.7
			TILTTTTIT	842-850	10.3
			LLVTKAVDIIIATT	854-867<br>848-861	82.5<br>31.6
			TAVLSTITSAV	894-904<br>888-889	17.4<br>17.9
			VSILSAVT	919-926<br>913-920	16.2<br>11.8
			SMVFVSTYT	1041-1049	67.4
			STVTATFSVT	1241-1250<br>1235-1244	16.9<br>16.4
			FLLSFIVLF	1296-1304<br>1290-1298	98.7
KGK38259.1*	Hypothetical protein with Flo11 domain	3.00E-05	PARVLVLAATNLP	343-349	87.96
